# Mathematical modeling of a MoSe₂-based SPR biosensor for detecting SARS-CoV-2 at nM concentrations

**DOI:** 10.3389/fbioe.2025.1547248

**Published:** 2025-02-28

**Authors:** Talia Tene, Nataly Bonilla García, Jessica Alexandra Marcatoma Tixi, Martha Ximena Dávalos Villegas, Cristian Vacacela Gomez, Stefano Bellucci

**Affiliations:** ^1^ Department of Chemistry, Universidad Técnica Particular de Loja, Loja, Ecuador; ^2^ Facultad de Ciencias, Escuela Superior Politécnica de Chimborazo (ESPOCH), Riobamba, Ecuador; ^3^ Carrera de Estadística, Escuela Superior Politécnica de Chimborazo (ESPOCH), Riobamba, Ecuador; ^4^ Carrera de Matemática, Escuela Superior Politécnica de Chimborazo (ESPOCH), Riobamba, Ecuador; ^5^ INFN-Laboratori Nazionali di Frascati, Frascati, Italy

**Keywords:** surface plasmon resonance, MoSe2, silicon nitride, SARS-CoV-2, biosensor, TMM approach

## Abstract

The rapid and accurate detection of SARS-CoV-2 remains a critical challenge in biosensing technology, necessitating the development of highly sensitive and selective platforms. In this study, we present a mathematical modeling approach to optimize a MoSe₂-based Surface Plasmon Resonance (SPR) biosensor for detecting the novel coronavirus at nM scale. Using the Transfer Matrix Method (TMM), we systematically optimize the biosensor’s structural parameters, including silver (Ag), silicon nitride (Si₃N₄), molybdenum diselenide (MoSe₂), and thiol-tethered single-stranded DNA (ssDNA) layers, to enhance sensitivity, detection accuracy, and optical performance. The results indicate that an optimized 45 nm Ag layer, 10 nm Si₃N₄ layer, and monolayer MoSe₂ configuration achieves a resonance shift (Δθ) of 0.3° at 100 nM, with a sensitivity of 197.70°/RIU and a detection accuracy of 5.24 × 10⁻^2^. Additionally, the incorporation of a 10 nm ssDNA functionalization layer significantly enhances molecular recognition, lowering the limit of detection (LoD) to 2.53 × 10⁻^5^ and improving overall biosensing efficiency. Sys₅ (MoSe₂ + ssDNA) outperforms Sys₄ (MoSe₂ without ssDNA) in terms of specificity and reliability, making it more suitable for practical applications. These findings establish the MoSe₂-based SPR biosensor as a highly promising candidate for SARS-CoV-2 detection, offering a balance between high sensitivity, optical stability, and molecular selectivity, crucial for effective viral diagnostics.

## 1 Introduction

The Coronavirus Disease 2019 (COVID-19) pandemic, caused by the Severe Acute Respiratory Syndrome Coronavirus 2 (SARS-CoV-2) (Kandi et al., 2021; Lai et al., 2020), has posed significant challenges to global healthcare systems. First identified in December 2019, the virus has since spread rapidly, leading to severe respiratory infections with symptoms ranging from mild flu-like effects to acute respiratory distress syndrome (ARDS) and multi-organ failure (Rabaan et al., 2023). SARS-CoV-2 primarily spreads via respiratory droplets, direct contact, and airborne transmission, infecting human cells through interaction with angiotensin-converting enzyme 2 (ACE2) receptors (Zuo et al., 2020). Early detection of the virus is crucial in controlling its transmission, ensuring timely medical intervention, and preventing large-scale outbreaks. Current diagnostic methods rely heavily on Reverse Transcription Polymerase Chain Reaction (RT-PCR) (Na et al., 2025), considered the gold standard for SARS-CoV-2 detection. However, RT-PCR requires specialized laboratory infrastructure, skilled personnel, and significant processing time, limiting its applicability in mass testing scenarios (Hassan et al., 2025). Rapid antigen-antibody tests provide faster results but often suffer from lower sensitivity and specificity. Alternative nucleic acid amplification techniques such as Loop-Mediated Isothermal Amplification (LAMP) (Augustine et al., 2020) offer a simpler and more rapid detection process, yet they still require additional steps and reagents. Given these limitations, there is an urgent need for real-time, highly sensitive, and label-free diagnostic techniques that can facilitate fast, accurate, and scalable viral detection.

Among emerging diagnostic tools, Surface Plasmon Resonance (SPR) biosensors have shown remarkable potential for real-time detection of biomolecules, including viruses (Takemura, 2021). SPR is an optical sensing technique that detects biomolecular interactions by measuring refractive index (RI) changes at the interface between a metallic thin film (typically silver (Tene et al., 2025a) or gold (Tene et al., 2024a) thin films) and a dielectric medium (e.g., a biological sample (Zhou et al., 2024)). When a biological molecule, such as a viral protein or antibody, binds to a functionalized SPR sensor surface, it induces a shift in the SPR resonance angle, which can be measured with high precision (Kushwaha et al., 2018). This technique provides a label-free, highly sensitive, and rapid method for detecting SARS-CoV-2 viral particles in clinical samples (Mousania et al., 2025; Tene et al., 2024b; Tene et al., 2024c). Unlike PCR-based techniques, SPR biosensors do not require amplification steps, making them a promising alternative for point-of-care applications.

Despite their advantages, traditional SPR biosensors face challenges in enhancing detection sensitivity and lowering the limit of detection (LoD). The integration of two-dimensional (2D) nanomaterials into SPR platforms has been explored as a means to significantly improve sensor performance (Tene et al., 2024d). Among 2D materials, graphene has been widely studied due to its high electrical conductivity, large surface area, and outstanding biocompatibility (Zhang et al., 2022; Tene et al., 2023). However, graphene’s moderate plasmonic enhancement limits its efficiency in improving SPR sensitivity. Recent studies have shown that transition metal dichalcogenides (TMDs) (Zhao et al., 2021), such as molybdenum diselenide (MoSe₂) (Anandh et al., 2024), exhibit superior optical, electronic, and plasmonic properties, making them an excellent alternative for next-generation SPR biosensors.

MoSe₂ is a layered semiconductor with strong plasmonic activity, a high refractive index, and an enhanced light-matter interaction (Yaremko et al., 2015), which makes it an ideal material for boosting SPR signal sensitivity. Unlike graphene, MoSe₂ possesses an intrinsic bandgap, allowing better control over its optical and electronic properties (Mishra et al., 2021). The incorporation of MoSe₂ into SPR biosensors results in stronger resonance shifts, improved refractive index sensitivity, and higher signal-to-noise ratio (SNR). Furthermore, when functionalized with Thiol-tethered ssDNA, MoSe₂ can serve as an efficient viral RNA detection platform, enhancing both specificity and binding affinity for SARS-CoV-2 biomarkers (Tene et al., 2025b).

Then, this study proposes a MoSe₂-based SPR biosensor for detecting SARS-CoV-2 with enhanced sensitivity and accuracy. Through numerical simulations using the Transfer Matrix Method (TMM) (Wu et al., 2010), we systematically analyze the effects of MoSe₂ layer thickness, silver (Ag) film properties, and ssDNA functionalization on SPR sensor performance. The study evaluates key biosensor performance metrics, including the SPR resonance angle shift, attenuation percentage, Full Width at Half Maximum (FWHM), sensitivity enhancement, Figure of Merit (FoM), Limit of Detection (LoD), and Signal-to-Noise Ratio (SNR). The goal is to optimize biosensor configurations to achieve maximum sensitivity to the refractive index change while minimizing noise and losses.

## 2 Materials and methods

### 2.1 Numerical modeling

Building on our previous studies (Tene et al., 2024a; Tene et al., 2024c; Tene et al., 2025b) and further supported by (Wu et al., 2010), this approach enables a systematic analysis of light propagation through a multilayer system by incorporating the optical properties and thickness of each layer. The layer thickness is defined along the perpendicular (z-axis), normal to the plane of the layers. Boundary conditions are applied at each interface to ensure continuity and accurately describe the interaction of electromagnetic waves across the structure. At the first layer (Z = Z_1_ = 0), the incident wave encounters the multilayer system, where its reflection and transmission coefficients are determined. The wave propagates through the layers until it reaches the final layer at Z = Z_n−1_, where *n* represents the total number of layers in the system. These boundary conditions enforce key physical constraints, such as energy conservation and phase continuity, across all interfaces.

The Z-coordinate represents the depth within the multilayer structure, with Z_1_ = 0 corresponding to the initial layer (e.g., the BK7 prism) and Z_n−1_ denoting the final layer (e.g., the ssDNA functionalization layer). TMM calculates the reflectance by constructing a matrix for each layer based on its refractive index, thickness, and incident angle. These matrices are sequentially combined using the boundary conditions to derive the overall reflectance and transmittance of the system. Then, the transfer matrix describes the relationship between the tangential components of the electric and magnetic fields:

$$\left[\begin{array}{c} E_{1}\\ H_{1} \end{array}\right]=M\left[\begin{array}{c} E_{N-1}\\ H_{N-1} \end{array}\right]$$ (1)

where, $E_{1}$ , $H_{1}$ , $E_{N-1}$ , and $H_{N-1}$ represent the tangential components of electric and magnetic fields at the first and last layer interfaces, respectively. M is represented by elements $M_{ij}$ as:

$$M=\prod_{k=2}^{N-1}M_{k}=\left[\begin{array}{cc} M_{11} & M_{12}\\ M_{21} & M_{22} \end{array}\right]$$ (2)

And $M_{k}$ is defined as:

$$M_{k}=\left[\begin{array}{cc} \cos\beta_{k} & -i\sin\beta_{k}/q_{k}\\ -iq_{k}\;\sin\beta_{k} & \cos\beta_{k} \end{array}\right]$$ (3)

here, k is an integer number. Additionally, $\beta_{k}$ is the phase thickness and $q_{k}$ is the refractive index in each layer:

$$\beta_{k}=\frac{2\pi d_{k}}{\lambda_{0}}\sqrt{\varepsilon_{k}-n_{1}^{2}\sin^{2}\theta }$$ (4)

And

$$q_{k}=\frac{\sqrt{\varepsilon_{k}-n_{1}^{2}\sin^{2}\theta }}{\varepsilon_{k}}$$ (5)

where, θ is the angle of incidence, $\lambda_{0}$ is the incident wavelength light, $n_{1}$ is the refractive index of the prism, $d_{k}$ is the thickness layer, and the local dielectric function $\varepsilon \left(\lambda_{0}\right)$ can be adopted as $n\left(\lambda_{0}\right)$ . Hence, the total reflection analysis of the N-layer system is obtained as:

$$R={\left|\frac{\left(M_{11}+M_{12}q_{N}\right)q_{1}-\left(M_{21}+M_{22}q_{N}\right)}{\left(M_{11}+M_{12}q_{N}\right)q_{1}+\left(M_{21}+M_{22}q_{N}\right)}\right|}^{2}$$ (6)

by using Equation 6 the SPR curve as a function of the angle of incidence is computed. To analyze the performance of the biosensor is necessary to consider the following metrics. The sensitivity of the biosensors ( S ) is defined as the multiplication of the sensitivity to the refractive index change ( $S_{RI}$ ) and the adsorption efficiency of the target analyte ( E ) as:

$$S=S_{RI}\cdot E$$ (7)

For biosensor optimization, we focus on the sensitivity enhancement ( $∆S_{RI}$ ) by optimizing each layer in water and PBS solutions, denoted as:

$$∆S_{RI}=\left(S_{RI}^{PBS}-S_{RI}^{0}\right)/S_{RI}^{0}$$ (8)

The sensitivity to the refractive index change can be expressed as:

$$S_{RI}=∆\theta /∆n$$ (9)

The parameter ∆θ represents the angle shift variation and ∆n is the change in refractive index. The detection accuracy (DA) can be written in terms of ∆θ and FWHM as:

DA=∆θ/FWHM (10)

Quality factor (QF) can be expressed in terms of S and FWHM as:

QF=S/FWHM (11)

In addition, to compute the FoM, LoD, and SNR, the related equations can be expressed as:

FoM=QF/Rmin (12)

$$\text{LoD}=\frac{∆n}{∆\theta }\times 0.005$$ (13)

$$\text{SNR}=\frac{∆\theta }{FWHM}$$ (14)

Where, Rmin is the resonance minimum from SPR curve and 0.005 is expressed in degree ( 0.005° ). All calculations in this study were conducted with a data sampling density of 50,000 points. This high-resolution sampling was selected to enhance precision, reduce numerical errors, and ensure a reliable foundation for the analysis.

### 2.2 Biosensor architecture

**FIGURE 1:**
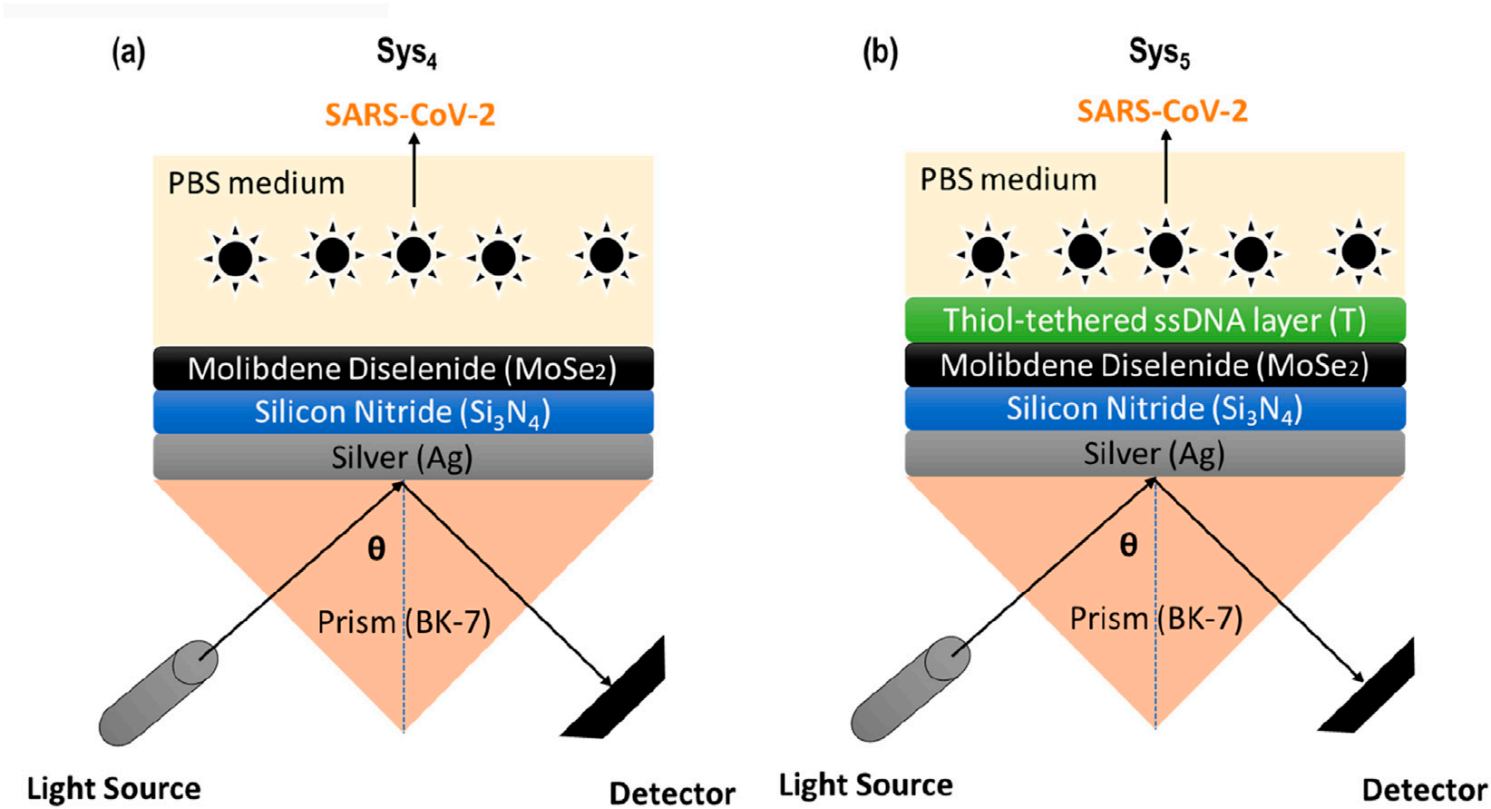
Schematic representation of the proposed SPR biosensor configurations (Sys₄ and Sys₅) for detecting SARS-CoV-2. **(A)** Sys₄ consists of a MoSe₂/Silicon Nitride/Silver multilayer structure, while **(B)** Sys₅ incorporates an additional Thiol-tethered ssDNA layer (T) for enhanced biomolecular interaction. The SPR response is analyzed using a BK-7 prism.

The construction of an efficient SPR biosensor relies on the precise selection and arrangement of functional layers, each contributing to the system’s overall sensitivity and stability. Figure 1 illustrates the structural arrangement of the proposed biosensors (selection criteria discussed below), while Table 1 summarizes the different configurations examined in this study. The biosensor design evolves a step-by-step approach, starting from the most basic setup (Sys₀) and progressively incorporating new layers that are expected to enhance the optical and sensing performance of the complete sensor. At the core of the sensor is the BK-7 prism, which serves as the optical medium for generating the SPR effect (Albelbeisi et al., 2024). Specifically, its high refractive index enables the total internal reflection (TIR) required to excite surface plasmons at the metal-dielectric interface. The angle at which light is coupled into the system is highly sensitive to refractive index changes near the sensor surface, making the prism an indispensable component in achieving real-time detection (Jaiswal et al., 2024).

**TABLE 1:** Configurations of SPR biosensor systems considered in this study. Sys₀ represents the basic setup with a Prism/Silver/PBS medium, while Sys₁ introduces SARS-CoV-2 in the PBS environment. From Sys₂ onward, additional layers (Si₃N₄, MoSe₂, and ssDNA) are incorporated to evaluate their impact on sensor performance under PBS + Virus conditions.

Sys no.	Code	Full Name	Notation
0	Sys_0_	Prism/Silver/PBS medium	P/Ag/M_PBS_
1	Sys_1_	Prism/Silver/PBS + SARS-CoV-2	P/Ag/M_PBS+Virus_
2	Sys_2_	Prism/Silver/Si_3_N_4_/PBS + SARS-CoV-2	P/Ag/SN/M_PBS+Virus_
3	Sys_3_	Prism/Silver/Si_3_N_4_/ssDNA/PBS + SARS-CoV-2	P/Ag/SN/T/M_PBS+Virus_
4	Sys_4_	Prism/Silver/Si_3_N_4_/Molybdenum Diselenide/PBS + SARS-CoV-2	P/Ag/SN/MoSe_2_/M_PBS+Virus_
5	Sys_5_	Prism/Silver/Si_3_N_4_/Molybdenum Diselenide/ssDNA/PBS + SARS-CoV-2	P/Ag/SN/MoSe_2_/T/M_PBS+Virus_

Directly above the prism lies the silver (Ag) layer, which is responsible for the excitation of surface plasmons. Silver is chosen over other noble metals, such as gold (Menon et al., 2018), due to its lower optical losses and sharper resonance curves, which enhance the SPR signal contrast and improve sensitivity (Oates et al., 2005). However, silver also presents some limitations, particularly in its susceptibility to oxidation and structural degradation over time. This makes the incorporation of protective and performance-enhancing layers essential to ensure long-term stability and reliable sensing capabilities. Then, the silicon nitride (Si₃N₄) layer, introduced in Sys₂ and subsequent configurations, plays a crucial role in overcoming some of silver’s limitations while also significantly improving the biosensor’s optical response (Kumar et al., 2022). As a high-refractive-index dielectric, Si₃N₄ enhances the evanescent field penetration depth, leading to improved light-matter interaction at the sensor surface. This property is particularly valuable in detecting small changes in refractive index, as it amplifies the resonance shift caused by molecular binding events. Additionally, the presence of Si₃N₄ helps to reduce metal-related propagation losses, improving signal stability and making the system more resistant to environmental variations. Another advantage of this layer is its chemical robustness (Mudgal et al., 2020), preventing direct interaction between the biological medium and the silver layer, which could otherwise degrade the sensor’s performance over time.

With this in mind, MoSe₂ is introduced in Sys₄ as an enhancement layer, further refining the biosensor’s ability to detect refractive index variations (Akib et al., 2024). Its strong light absorption and plasmonic interaction improve the resonance shift, leading to increased sensitivity to biomolecular interactions (Haque and Rouf, 2021). This enhancement arises from MoSe₂'s high refractive index and excitonic effects, which strengthen the coupling between the incident light and the plasmonic resonance. Furthermore, MoSe₂ contributes to improved confinement of the electromagnetic field, particularly at the metal-dielectric interface, thereby enhancing the field intensity within the sensing region. This tighter confinement increases the sensor’s responsiveness to small variations in the surrounding medium, making it more effective in detecting subtle changes in biomolecular binding (Rouf and Haque, 2021). Additionally, the anisotropic optical properties of MoSe₂, such as its strong in-plane and out-of-plane permittivity contrast, aid in optimizing the resonance conditions and reducing propagation losses, further improving the sensor’s overall performance (Haque and Rouf, 2021; Rouf and Haque, 2021).

The final structural modification, introduced in Sys₅, involves the thiol-tethered ssDNA functionalization. This layer is designed to enable specific biomolecular recognition, enhancing the sensor’s selectivity for SARS-CoV-2 detection. By forming stable covalent bonds with MoSe₂, the ssDNA layer ensures that only target viral RNA sequences bind effectively, minimizing false positives and improving overall detection accuracy (Lee et al., 2008; Kaur et al., 2016).

## 3 Results and discussions

The values presented in Supplementary Table S1 summarize the refractive indices (RI) and thicknesses of the materials used in the construction of the MoSe₂-based SPR biosensor. These parameters, sourced from previous theoretical and experimental studies, serve to optimize the biosensor’s performance. The RI values at 633 nm have been carefully chosen to ensure that each layer contributes effectively to the light-matter interactions necessary for highly sensitive SPR sensing. The dielectric media, including water, phosphate-buffered saline (PBS), and SARS-CoV-2-containing samples, are also characterized by their respective refractive indices. Notably, the PBS medium (Sys₀) has an RI of 1.334, which represents the base system for the initial simulations. However, for systems designed to detect SARS-CoV-2, we assume an RI of 1.340, corresponding to a viral concentration of 150 mM, as experimentally reported in Ref. (Kumar et al., 2022). An important consideration when analyzing these values is that while the RI of SARS-CoV-2 at 150 mM provides a benchmark for biosensor performance, it may not be entirely realistic for clinical applications, where viral loads are typically much lower. This is particularly relevant when considering realistic diagnostic settings, where virus concentrations are usually in the nanomolar range (nM, i.e., 10^9^–10^13^ particles/mL (Tene et al., 2025a)). Recognizing this, our study will first optimize the biosensor layer-by-layer using the fixed refractive index values from Supplementary Table S1, ensuring that the configurations are optimized under controlled conditions. After obtaining the best-performing configurations, we will further evaluate their performance at very low viral concentrations.

Hence, the RI of MoSe₂ (4.62 + 1.0063i) at 633 nm highlights its strong absorptive and refractive properties (Akib et al., 2024), making it an ideal plasmonic enhancement layer. Similarly, the Si₃N₄ layer (RI = 2.0394 (Kumar et al., 2022)) was selected for its ability to stabilize the silver layer and improve field confinement. The ssDNA functionalization (RI = 1.462 (Kumar et al., 2022)) ensures that the biosensor is biologically selective while maintaining strong optical properties. The refractive indices of PBS and water, which serve as the background medium in most experimental conditions, are close to each other (1.334 and 1.330, respectively), ensuring minimal baseline signal interference. However, the introduction of SARS-CoV-2 particles alters the effective refractive index of the medium, and the biosensor’s ability to detect such changes is central to its performance evaluation.

### 3.1 Configurations under investigation

**FIGURE 2:**
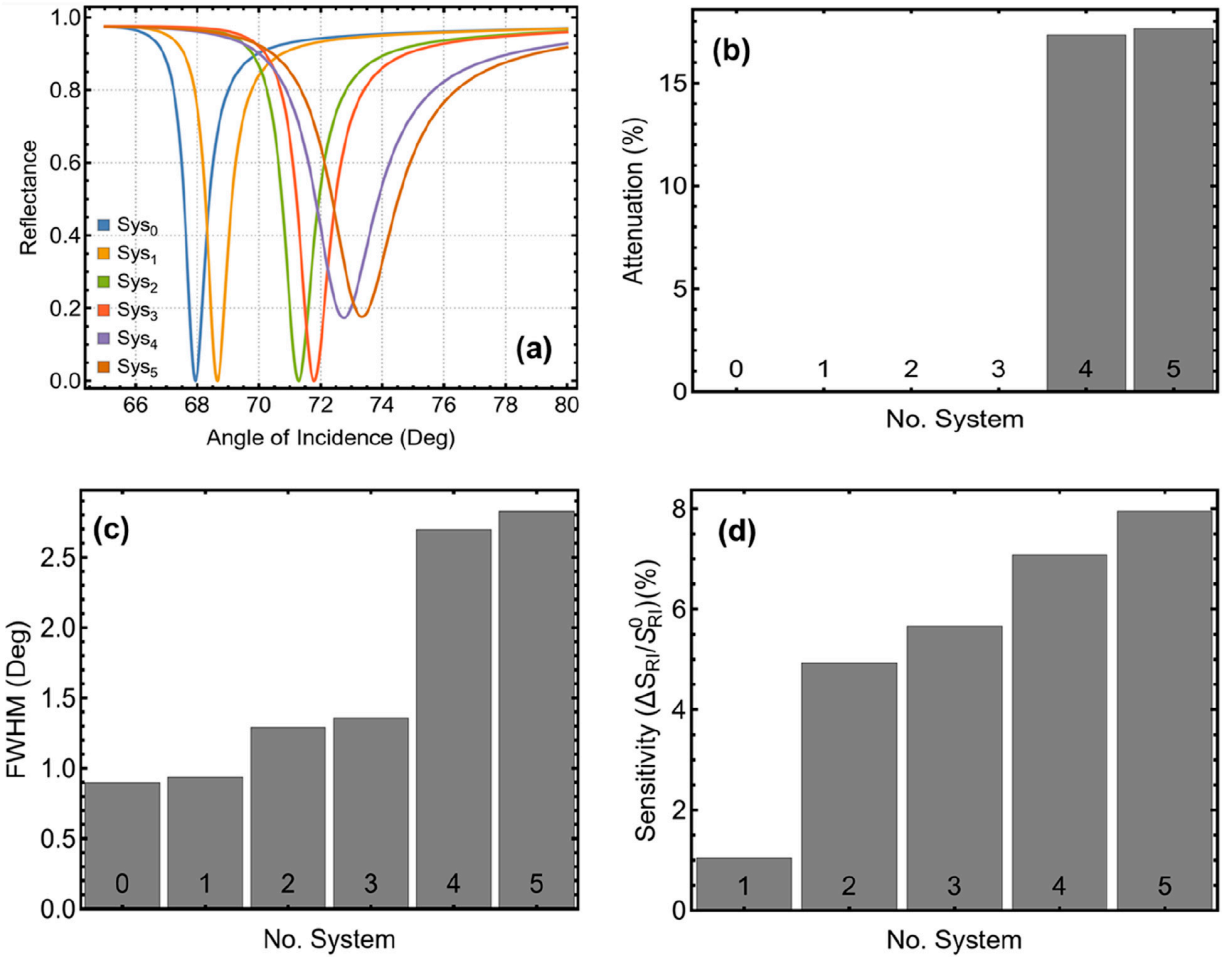
Performance analysis of the SPR biosensor configurations from Table 1. **(A)** Reflectance curves for each system, illustrating the SPR dip shifts. **(B)** Attenuation percentage, indicating the energy loss at resonance. **(C)** FWHM shows the sharpness of the resonance dip. **(D)** Sensitivity enhancement relative to Sys₀ (PBS-only system). Sys₄ and Sys₅ exhibit the most promising results regarding sensitivity, FWHM, and attenuation, making them the optimal configurations for further analysis.

The results presented in Figure 2 and Supplementary Table S2 demonstrate how the introduction of additional functional layers in the SPR biosensor impacts key performance parameters, including the SPR peak position, attenuation, FWHM, and sensitivity enhancement. The progressive incorporation of Si₃N₄, MoSe₂, and ssDNA leads to significant improvements in the sensor’s ability to detect refractive index changes, ultimately allowing the selection of the most promising configurations for SARS-CoV-2 detection. The reflectance curves (obtained by Equation 6) in Figure 2A reveal a clear shift in the SPR dip position as new layers are added. The base configuration (Sys₀ in PBS) exhibits an SPR peak at 67.94°, while the introduction of the virus (Sys₁) results in a slight increase to 68.65° due to the refractive index change from PBS to PBS + Virus. As additional layers are incorporated, this peak progressively shifts toward higher angles, reaching 72.76° for Sys₄ and 73.34° for Sys₅, as shown in Supplementary Table S2. This shift is a direct consequence of the increasing optical path length and enhanced plasmonic interaction, reinforcing the idea that MoSe₂ and ssDNA contribute to stronger refractive index sensitivity.

The attenuation percentage, depicted in Figure 2B and quantified in Supplementary Table S2, remains negligible for Sys₀ to Sys₃, with values below 0.01%. However, a substantial increase occurs in Sys₄ (17.33%) and Sys₅ (17.64%), which is expected given the additional material layers. While higher attenuation generally implies greater energy loss, in this case, it also indicates stronger light-matter interaction and enhanced plasmonic excitation, which are favorable for sensing applications. Nonetheless, excessive attenuation could compromise detection accuracy, necessitating a balance between signal strength and energy loss. A similar trend is observed for FWHM, illustrated in Figure 2C. The baseline configuration (Sys₀) has the narrowest FWHM (0.90°), while Sys₅ exhibits the broadest value (2.83°). A wider resonance dip can reduce precision in detecting small refractive index changes, but it also indicates a higher coupling efficiency between incident light and surface plasmons. Thus, Sys₄ and Sys₅ strike a balance between sharp resonance and sufficient energy coupling, making them ideal for biosensing applications.

The sensitivity enhancement (by Equation 8), shown in Figure 2D, highlights the superior performance of Sys₄ and Sys₅ compared to other configurations. While Sys₂ and Sys₃ already show a notable improvement over the baseline, the incorporation of MoSe₂ (Sys₄) and the additional ssDNA layer (Sys₅) further amplifies the sensor’s response. The highest enhancement is observed in Sys₅ (7.95%), confirming the positive effect of functionalization in improving the biosensor’s specificity toward SARS-CoV-2. Taken together, these results support the selection of Sys₄ and Sys₅ as the most effective configurations for further investigation (see Figure 1). They exhibit a strong resonance shift, controlled attenuation, and enhanced sensitivity, making them highly suitable for detecting subtle refractive index changes associated with viral adsorption.

### 3.2 Optimization: silver thickness

**FIGURE 3:**
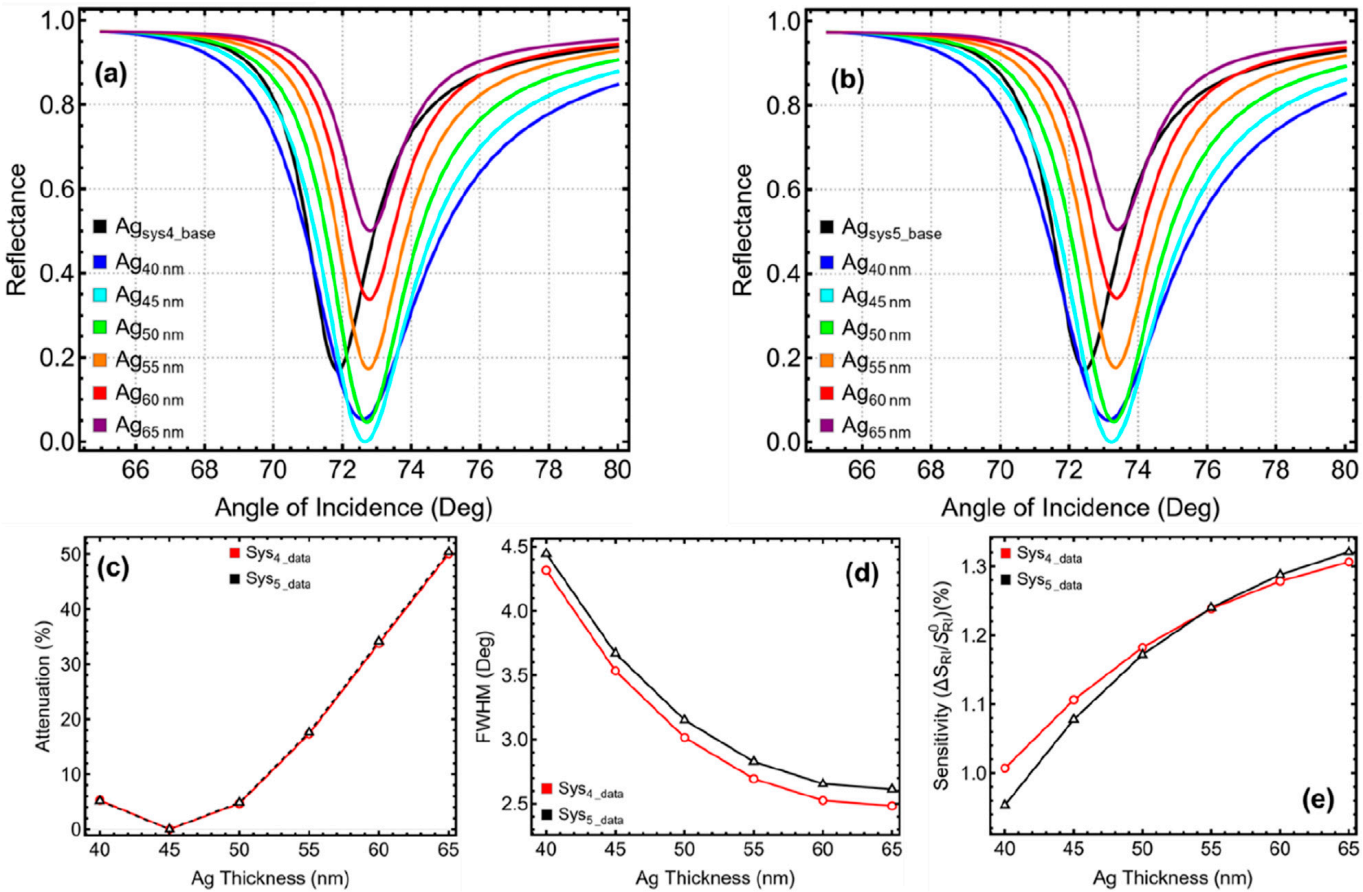
Optimization of silver (Ag) layer thickness for Sys₄ and Sys₅. **(A)** SPR reflectance curves for Sys₄ with varying Ag thickness (40–65 nm), compared to the unoptimized Ag_Sys4_base_ configuration. **(B)** SPR reflectance curves for Sys₅ under the same conditions, compared to Ag_Sys5_base_. **(C)** Attenuation percentage as a function of Ag thickness. **(D)** FWHM shows the narrowing effect of thinner Ag layers. **(E)** Sensitivity enhancement relative to unoptimized systems.

The optimization of the silver (Ag) layer thickness plays a crucial role in achieving a balance between sensitivity, resonance sharpness, and energy attenuation. The results presented in Figure 3 and Supplementary Table S3 demonstrate how varying the Ag thickness from 40 to 65 nm influences key performance parameters, including the SPR peak position, attenuation, FWHM, and sensitivity enhancement for Sys₄ and Sys₅. The SPR reflectance curves in Figures 3A, B show that decreasing the Ag thickness results in a slight shift in the resonance angle, confirming that the optical path length is influenced by the metallic layer thickness. However, the most notable changes are observed in attenuation. The attenuation percentage, shown in Figure 3C, remains relatively low for thinner Ag layers but increases significantly beyond 55 nm, reaching extremely high values at 60 nm and 65 nm (33.78% and 50.05% for both configurations, respectively). This excessive attenuation at higher thicknesses indicates that a substantial amount of light is being absorbed or scattered, leading to undesirable energy losses that could compromise detection accuracy.

At the same time, the FWHM values in Figure 3D show a decreasing trend as the Ag thickness increases. A thicker Ag layer narrows the SPR resonance dip, improving peak sharpness and spectral resolution. However, this comes at the cost of higher energy losses and reduced plasmonic coupling efficiency. Conversely, when the Ag layer is very thin (40 nm), the resonance dip becomes broad, leading to weaker confinement of surface plasmons. This results in a less-defined sensing signal with reduced sensitivity to refractive index changes. The sensitivity enhancement, illustrated in Figure 3E, confirms that thinner Ag layers contribute to a stronger sensor response, with values increasing as the thickness is reduced. However, beyond 45 nm, the improvement in sensitivity becomes marginal, while attenuation begins to rise significantly. This trade-off highlights the need to choose an optimal silver thickness that ensures sufficient plasmonic excitation without excessive losses.

Based on these observations, a silver thickness of 45 nm is selected for both Sys₄ and Sys₅, as it provides the best balance between FWHM sharpness, sensitivity enhancement, and controlled attenuation. As seen in Supplementary Table S3, at 45 nm, the attenuation remains negligible (0.003% for Sys₄ and Sys₅), while FWHM maintains a reasonable sharpness (3.53° for Sys₄ and 3.67° for Sys₅). Moreover, the sensitivity enhancement remains higher than the unoptimized configurations, ensuring that the biosensor maintains high sensitivity while minimizing energy losses. In addition to the thickness of the silver (Ag) layer, its morphology and crystallinity are critical factors that influence the performance of the SPR biosensor. A smooth, continuous, and highly crystalline Ag film is essential to ensure efficient surface plasmon excitation while minimizing scattering losses (Oates et al., 2005). Surface roughness, grain boundaries, and void formation can introduce plasmon damping effects, which lead to reduced sensitivity and an increased signal-to-noise ratio in the biosensor response.

### 3.3 Optimization: silicon nitride thickness

**FIGURE 4:**
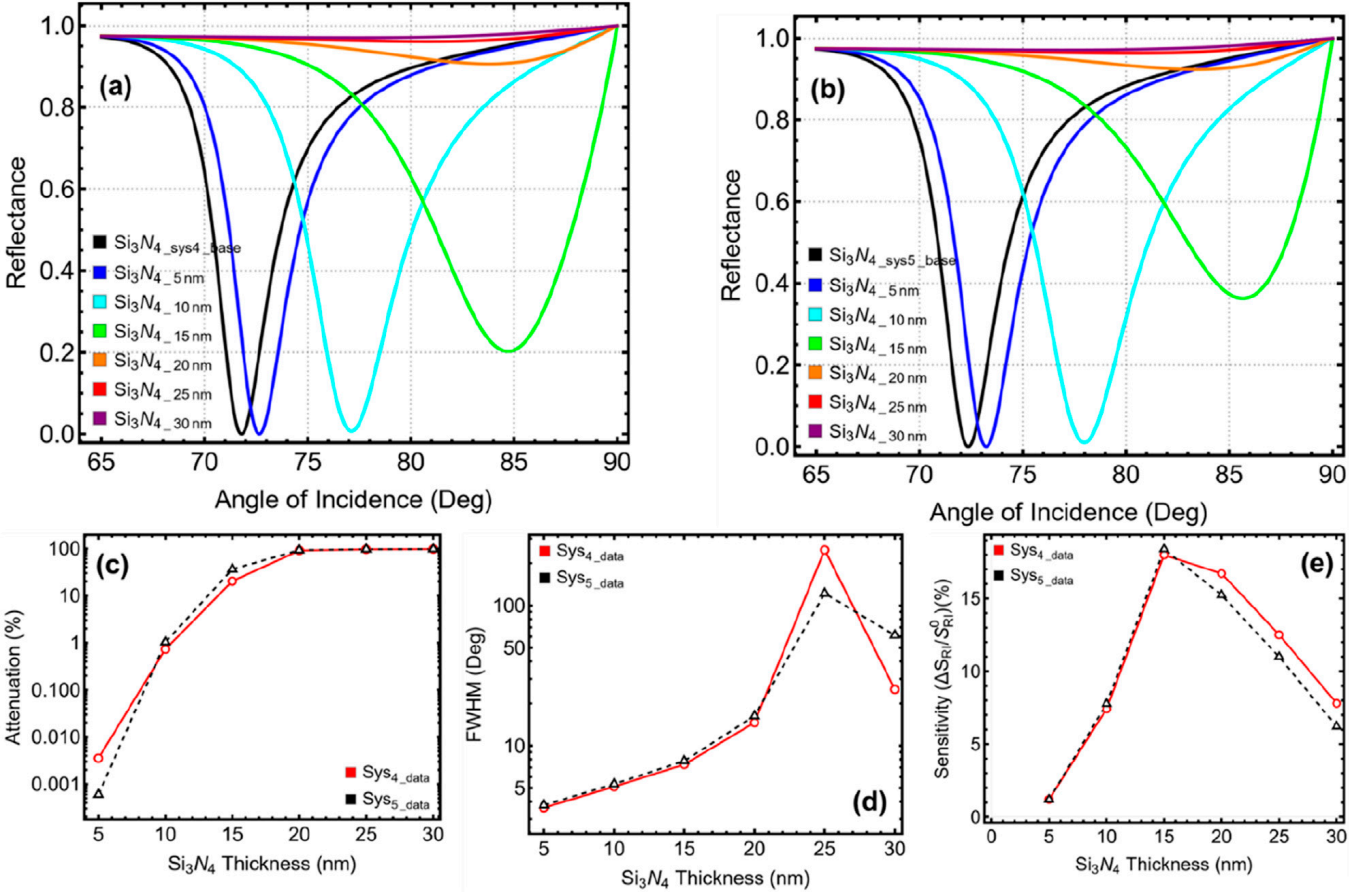
Optimization of silicon nitride (Si_3_N_4_) layer thickness for Sys₄ and Sys₅. **(A)** SPR reflectance curves for Sys₄ with varying Si_3_N_4_ thickness (5–30 nm), compared to the unoptimized Si_3_N_4_Sys4_base_ configuration. **(B)** SPR reflectance curves for Sys₅ under the same conditions, compared to Si_3_N_4_Sys5_base_. **(C)** Attenuation percentage as a function of Si_3_N_4_ thickness. **(D)** FWHM shows the effect of Si_3_N_4_ layer thickness. **(E)** Sensitivity enhancement relative to unoptimized systems.

The results presented in Figure 4 and Supplementary Table S4 demonstrate how varying the Si₃N₄ thickness from 5 nm to 30 nm affects the overall sensor response for Sys₄ and Sys₅. The reflectance curves in Figures 4A, B reveal a significant shift in the SPR peak position as the Si₃N₄ thickness increases. This shift is more pronounced beyond 15 nm, indicating that thicker Si₃N₄ layers introduce stronger refractive index variations at the interface. However, these large shifts come at the cost of excessive attenuation and broadening of the resonance dip, as seen in Figures 4C, D. The attenuation percentage, displayed in Figure 4C and quantified in Supplementary Table S4, remains negligible at 5 nm and 10 nm, but increases drastically beyond 15 nm, reaching extreme values at 20 nm and higher (90.63% and 97.06% for both systems, respectively). Such high attenuation levels indicate substantial energy loss, which would significantly reduce the biosensor’s detection efficiency.

A similar pattern is observed for FWHM, illustrated in Figure 4D. While a moderate increase in Si₃N₄ thickness improves plasmonic coupling, excessive thickness results in uncontrolled resonance broadening, ultimately degrading detection precision. The FWHM values at 20 nm and beyond are excessively high, with Sys₄ reaching 249.28° and Sys₅ reaching 121.81° at 25 nm, which are far too broad for an effective sensing signal. The sensitivity enhancement, presented in Figure 4E, initially increases with Si₃N₄ thickness, peaking around 15 nm before declining at higher thicknesses. The decline at 20 nm and above can be attributed to excessive damping effects, where the sensor’s response becomes less efficient due to the increased optical path length and reduced plasmonic confinement. Based on these trends, an Si₃N₄ thickness of 10 nm is selected as the optimal configuration for both Sys₄ and Sys₅. At 10 nm, the attenuation remains low (0.733% for Sys₄ and 1.033% for Sys₅), while the FWHM values are still within an acceptable range (5.11° for Sys₄ and 5.33° for Sys₅). Additionally, the sensitivity enhancement is significantly improved compared to the unoptimized case (7.42% for Sys₄ and 7.77% for Sys₅), making this the most balanced choice.

### 3.4 Optimization: MoSe_2_ layers

**FIGURE 5:**
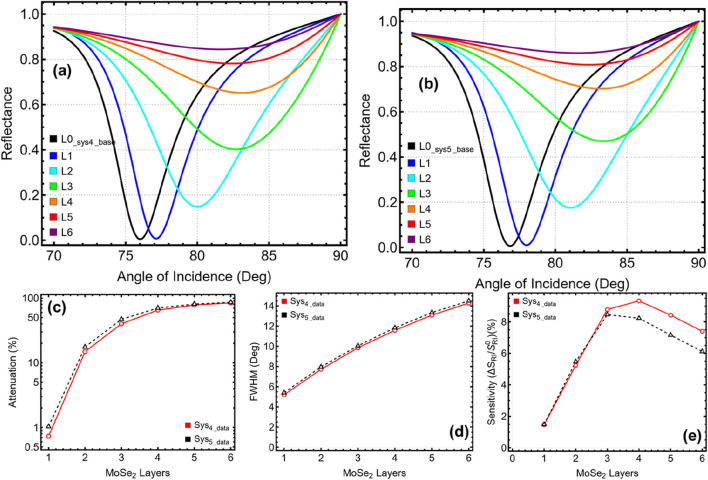
Optimization of Molybdenum Diselenide (MoSe_2_) layer thickness for Sys₄ and Sys₅. **(A)** SPR reflectance curves for Sys₄ with varying the number of MoSe_2_ layers (L1–L6), compared to the unoptimized L0__Sys4_base_ configuration. **(B)** SPR reflectance curves for Sys₅ under the same conditions, compared to L0__Sys5_base_. **(C)** Attenuation percentage as a function of the number of MoSe_2_ layers. **(D)** FWHM shows the effect of the number of MoSe_2_ layers. **(E)** Sensitivity enhancement relative to unoptimized systems.

The integration of MoSe₂ into the SPR biosensor enhances plasmonic resonance strength and sensing performance, but the optimal number of layers must be carefully chosen to balance sensitivity, attenuation, and spectral sharpness. The results presented in Figure 5 and Supplementary Table S5 evaluate the performance of Sys₄ and Sys₅ as the number of MoSe₂ layers is varied from 1 to 6 layers. The SPR reflectance curves in Figures 5A, B show a progressive shift in the resonance dip with increasing MoSe₂ layers. This shift is accompanied by a gradual increase in attenuation, as seen in Figure 5C. The attenuation is minimal for L1 (single-layer MoSe₂), with values of 0.733% for Sys₄ and 1.033% for Sys₅. However, attenuation increases drastically beyond L3, reaching 84.39% for Sys₄ and 85.94% for Sys₅ at L6, indicating that excessive MoSe₂ thickness causes significant energy loss, reducing the efficiency of plasmonic coupling.

A similar trend is observed for FWHM, as illustrated in Figure 5D. The resonance broadens significantly with additional MoSe₂ layers, reaching 14.29° for Sys₄ and 14.50° for Sys₅ at L6. Such broadening is undesirable, as it reduces spectral resolution, making it harder to detect small refractive index changes. The ideal sensor configuration should maintain a narrower FWHM, ensuring clear and well-defined resonance dips.

The sensitivity enhancement, presented in Figure 5E, initially improves as the number of MoSe₂ layers increases, peaking at L3 before declining at L4 and beyond. While a higher number of layers leads to greater sensitivity, this improvement is accompanied by a steep increase in attenuation and FWHM, reducing the overall efficiency of the biosensor. Considering these trade-offs, a single-layer MoSe₂ (L1) configuration is selected as the optimal design, as it maintains low attenuation, manageable FWHM, and reasonable sensitivity enhancement. Although this choice results in lower sensitivity compared to multilayer MoSe₂, it provides a practical and experimentally feasible approach while avoiding excessive plasmonic damping.

The fabrication of single-layer MoSe₂ can be achieved using various synthesis techniques, ensuring compatibility with the proposed biosensor. Common methods include chemical vapor deposition (CVD) (Wang et al., 2014), mechanical exfoliation (Liu et al., 2018), and molecular beam epitaxy (MBE) (Roy et al., 2016). Among these, CVD is the most scalable technique, allowing for controlled growth of uniform MoSe₂ monolayers on large-area substrates. Mechanical exfoliation, although widely used in fundamental few-layer graphene research (Vacacela Gomez et al., 2021), has limitations in terms of reproducibility and scalability. MBE, on the other hand, offers precise thickness control but is more complex and expensive. Regarding stability, MoSe₂ exhibits greater resistance to oxidation compared to its sulfur-based counterpart, MoS₂, due to the stronger Se-Mo bonding and lower reactivity of selenium in ambient conditions. However, prolonged exposure to air and humidity can still lead to gradual degradation, particularly in the presence of strong oxidative agents. To mitigate this, encapsulation strategies such as hexagonal boron nitride (h-BN) capping, polymer coatings, or integration with protective dielectric layers can be employed to preserve the structural and optical properties of MoSe₂ over time (Scuri et al., 2018; Shen et al., 2019). Actually, in this study, silicon nitride (Si₃N₄) is incorporated into the biosensor configuration (BK7/silver thin film/Silicon nitride/MoSe₂/ssDNA) to serve as both a protective layer and an optical enhancer (Kumar et al., 2022). Si₃N₄ provides excellent resistance against oxidation and moisture, ensuring the long-term stability of MoSe₂. These considerations enhance the reliability of the biosensor under operational conditions.

### 3.5 Optimization: thiol-tethered ssDNA thickness

**FIGURE 6:**
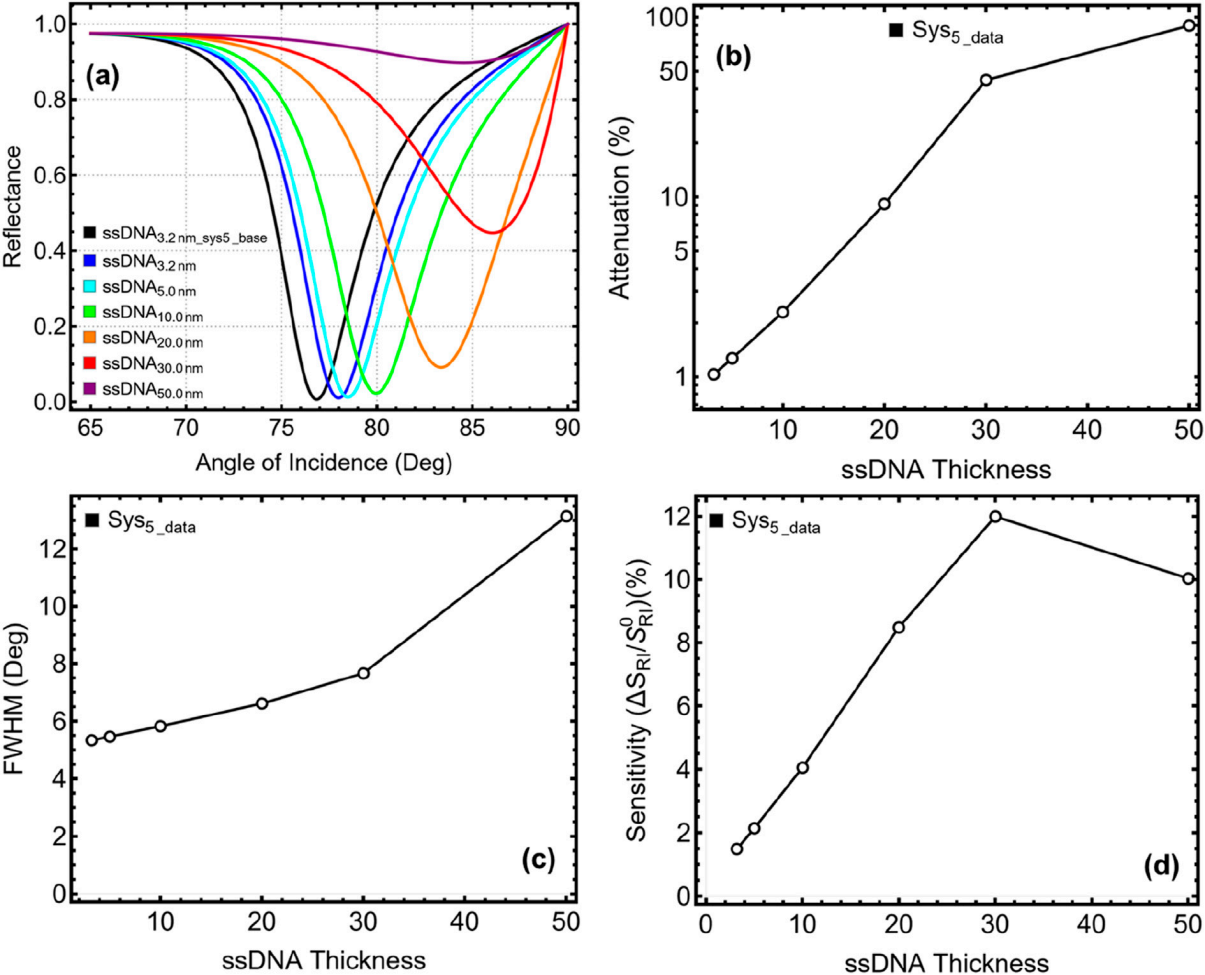
Optimization of ssDNA layer thickness for Sys₅. **(A)** SPR reflectance curves for different ssDNA thicknesses, compared to the unoptimized ssDNA_3.2nm_Sys5_base_ configuration. **(B)** Attenuation percentage as a function of ssDNA thickness. **(C)** FWHM shows the broadening effect as the ssDNA layer increases. **(D)** Sensitivity enhancement relative to the unoptimized system.

Optimizing the ssDNA thickness in Sys₅ is crucial for maintaining high specificity toward SARS-CoV-2 RNA while ensuring an efficient plasmonic response. The results in Figure 6 and Supplementary Table S6 analyze how varying the ssDNA thickness from 3.2 nm to 50 nm affects sensor performance. The SPR reflectance curves in Figure 6A indicate that as the ssDNA thickness increases, the resonance angle shifts toward higher values due to the increase in the effective refractive index at the sensing interface. This shift is quantified in Supplementary Table S6, where the SPR peak moves from 77.98° at 3.2 nm to 86.06° at 30 nm, demonstrating the strong optical modulation effect of thicker ssDNA layers. The attenuation percentage, shown in Figure 6B, remains low at thinner ssDNA layers but increases steeply beyond 10 nm. At 3.2 nm, attenuation is only 1.03%, while at 10 nm, it remains within an acceptable range at 2.29%. However, at 30 nm, attenuation reaches 44.71%, and at 50 nm, it surges to 89.73%, meaning that most of the light energy is being absorbed or scattered. Such high losses degrade the resonance signal and limit the sensor’s ability to detect minor refractive index variations effectively.

A similar pattern is observed in FWHM broadening, as shown in Figure 6C. Narrow resonance peaks are essential for high measurement precision, and while 5.33° at 3.2 nm and 5.82° at 10 nm maintain clear resonance features, further increasing the ssDNA thickness causes excessive broadening. At 50 nm, FWHM expands to 13.14°, reducing the ability to track subtle resonance shifts accurately. The sensitivity enhancement, presented in Figure 6D, improves with increasing ssDNA thickness up to 30 nm (11.99%) before slightly declining at 50 nm (10.03%). While greater sensitivity is beneficial, it must be weighed against attenuation and peak-broadening effects. The significant losses at 30 nm and beyond make those configurations impractical, despite their improved sensitivity.

Considering all these factors, 10 nm emerges as the best choice for the ssDNA layer thickness. This configuration keeps attenuation low, FWHM sharp, and sensitivity at a reasonable level, ensuring a well-balanced and experimentally feasible biosensor design. In particular, achieving stable ssDNA coatings below 5 nm presents fabrication challenges, as ultra-thin layers often suffer from inhomogeneities and reduced hybridization efficiency. In contrast, a 10 nm ssDNA layer can be reliably produced using established surface chemistry techniques, such as thiol-gold functionalization and layer-by-layer deposition, ensuring uniformity and stable biomolecular interactions (Kang et al., 2014).

### 3.6 Optimized biosensors: Sys_4_ and Sys_5_

The results presented in Table 2 summarize the final optimized configurations of Sys₄ and Sys₅, which will now be tested for SARS-CoV-2 detection at nanomolar (nM) concentrations. These refined parameters ensure that both biosensors achieve high sensitivity, strong plasmonic resonance, and minimal attenuation, making them suitable candidates for realistic virus sensing applications. Both configurations utilize BK-7 as the optical coupling medium, with a refractive index of 1.5151, which is crucial for efficient excitation of surface plasmons. The silver (Ag) layer, optimized at 45 nm, maintains a balance between plasmonic enhancement and optical losses, ensuring that the resonance dip remains sharp and well-defined for accurate sensing. The Si₃N₄ dielectric layer, kept at 10 nm, improves the field confinement and refractive index sensitivity while preventing excessive broadening of the resonance peak. The incorporation of MoSe₂ in both Sys₄ and Sys₅ as a monolayer (L = 1, thickness = 0.70 nm) leverages its strong optical properties, boosting the system’s ability to detect the refractive index variations. In Sys₅, an additional 10 nm ssDNA layer is introduced to facilitate specific binding interactions with SARS-CoV-2 RNA, enhancing the sensor’s selectivity. The next phase of the study involves testing Sys₄ and Sys₅ across different SARS-CoV-2 concentrations, evaluating their performance in accurately detecting viral presence at clinically relevant levels.

**TABLE 2:** Optimized parameters of Sys_4_ and Sys_5_ configurations.

Material	Refractive index (RI)	Thickness (nm)
Sys_4_		
BK7 (P)	1.5151	---
Ag	0.056253 + 4.2760	45.0
Si_3_N_4_ (SN)	2.0394	10.0
Molybdenum Diselenide	4.62 + 1.0063 i	0.70 (L = 1)
Sys_5_		
BK7 (P)	1.5151	---
Ag	0.056253 + 4.2760	45.0
Si_3_N_4_ (SN)	2.0394	10.0
Molybdenum Diselenide	4.62 + 1.0063 i	0.70 (L = 1)
ssDNA (Thiol-Tethered, T)	1.462	10.0

### 3.7 Optimized biosensors for viral detection

**FIGURE 7:**
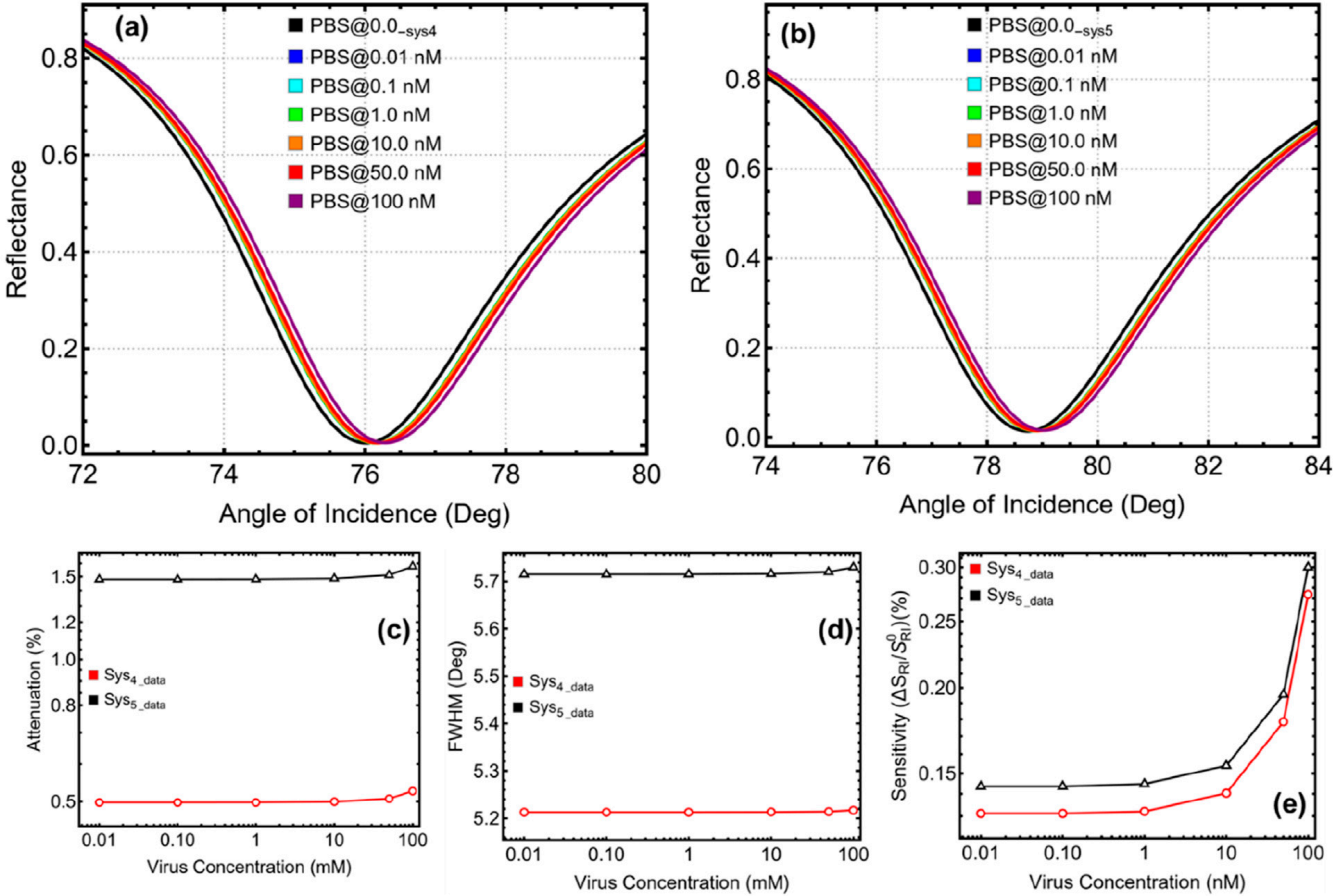
Evaluation of optimized Sys₄ and Sys₅ biosensors for SARS-CoV-2 detection across different viral concentrations (0.01 nM–100 nM). **(A)** SPR reflectance curves for Sys₄, and **(B)** SPR reflectance curves for Sys₅, comparing their responses to increasing virus concentrations. **(C)** Attenuation percentage as a function of viral concentration. **(D)** FWHM, indicating resonance broadening effects. **(E)** Sensitivity enhancement relative to the optimized PBS-only configurations (PBS@0.0__Sys4_) and PBS@0.0__Sys5_).

The results presented in Figure 7 and Supplementary Table S7 evaluate the performance of the optimized Sys₄ and Sys₅ biosensors in detecting SARS-CoV-2 at nanomolar (nM) concentrations, ranging from 0.01 nM to 100 nM. Given that the experimental RI values reported in (Kumar et al., 2022) were unsuitable for clinical applications, we relied on the data from (Akib et al., 2024) and applied a linear fit to estimate RI values for the chosen concentration range. This range closely aligns with clinically relevant viral loads, spanning approximately 6 × 10^9^ (0.01 nM) to 6 × 10^13^ (100 nM) particles/mL, allowing for a more realistic evaluation of the biosensors. To further contextualize, by considering 1 PFU = 1000 particles, these viral concentrations are found between 6 × 10^6^ PFU/mL (0.01 nM) and 6 × 10^10^ PFU/mL (100 nM).

The SPR reflectance curves in Figures 7A, B demonstrate a gradual shift in the resonance angle as virus concentration increases, confirming the biosensors’ ability to detect the refractive index variations induced by viral adsorption. However, the magnitude of these shifts is relatively small (see Supplementary Figure S1), which is expected given the low refractive index contrast introduced by nanomolar viral concentrations. The numerical values in Supplementary Table S7 further support this trend, with Sys₄‘s resonance peak shifting from 78.69° at 0.1 nM to 78.86° at 100 nM, while Sys₅‘s peak moves from 83.16° at 0.1 nM to 83.40° at 100 nM. This confirms that both configurations remain sensitive to viral presence even at low concentrations.

The attenuation percentages, displayed in Figure 7C, remain relatively low, ensuring that the biosensors maintain a strong signal. In Sys₄, attenuation increases from 0.50% at 0.1 nM to 0.53% at 100 nM, whereas in Sys₅, it rises from 1.48% to 6.30% over the same range. The slightly higher attenuation in Sys₅ is likely due to the ssDNA functionalization layer, which facilitates stronger virus-RNA interactions, slightly increasing energy absorption. Nonetheless, the values remain well within an acceptable range, preserving signal integrity for accurate detection.

The FWHM values, shown in Figure 7D, indicate minimal resonance broadening, with Sys₄ varying only from 5.21° to 5.22° and Sys₅ from 5.72° to 5.73°. This confirms that the introduction of viral particles does not significantly degrade the sharpness of the resonance peak, which is crucial for precise refractive index detection. Meanwhile, sensitivity enhancement, as illustrated in Figure 7E, increases as virus concentration rises, reaching 0.36% in Sys₄ and 0.55% in Sys₅ at 100 nM. Although these sensitivity values are lower than those observed during previous optimization steps, this is expected given that nanomolar concentrations introduce only small refractive index changes.

Despite the similar numerical results between Sys₄ and Sys₅, the absence of the ssDNA layer in Sys₄ presents a critical limitation. While both configurations detect changes in the refractive index due to viral presence, Sys₄ lacks the biochemical specificity necessary for direct SARS-CoV-2 RNA sensing. The presence of ssDNA in Sys₅ significantly improves selectivity, as it facilitates targeted hybridization with viral RNA sequences, ensuring more reliable and specific detection. Without this layer, Sys₄ relies on non-specific interactions between the virus and the MoSe₂/Si₃N₄ interface, which could lead to false positives or reduced efficiency in distinguishing SARS-CoV-2 from other biological components.

### 3.8 Biosensor performance

**FIGURE 8:**
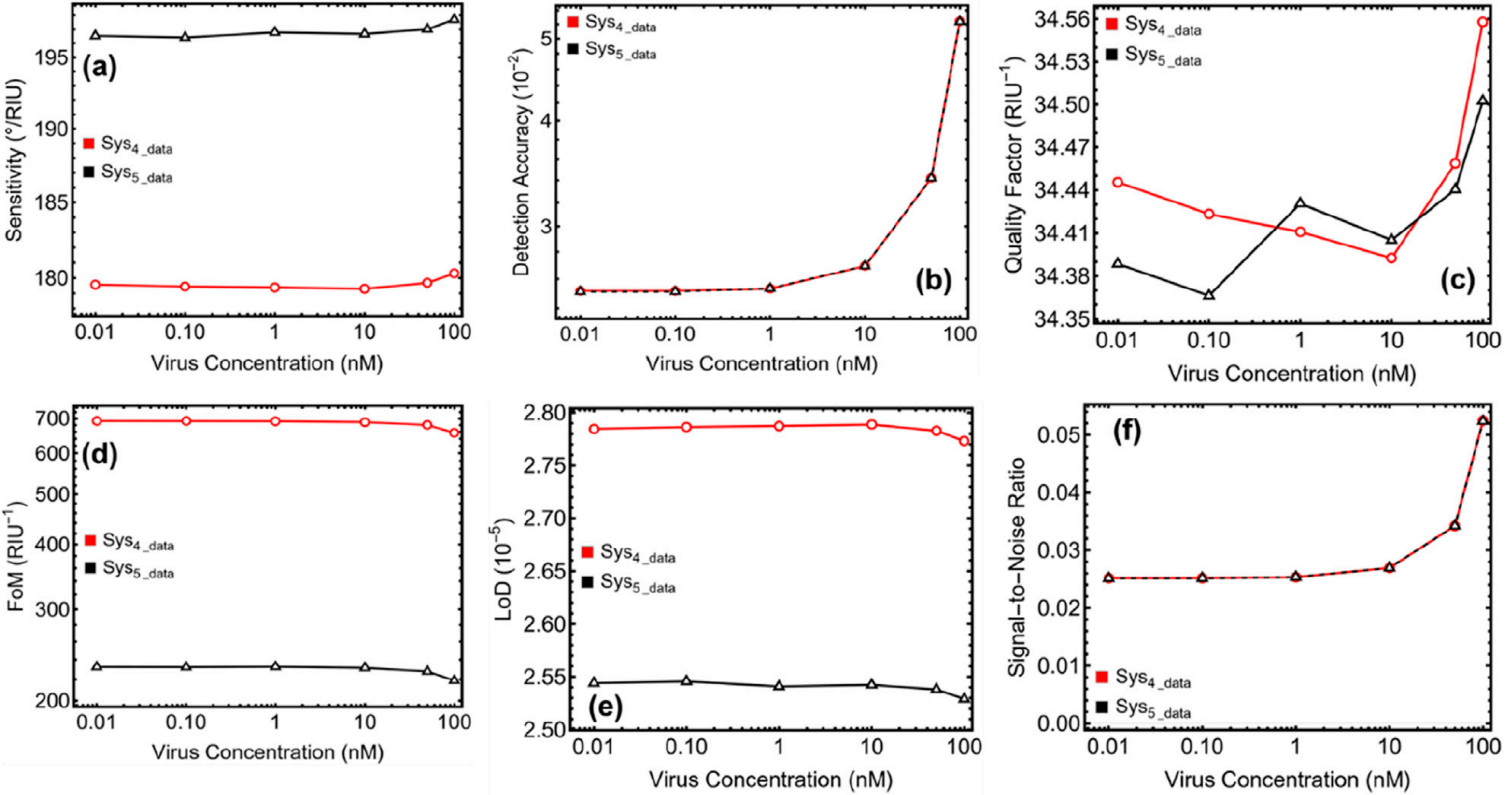
Performance evaluation of optimized Sys₄ and Sys₅ biosensors against SARS-CoV-2, analyzing key metrics for biosensing efficiency. **(A)** Sensitivity to refractive index change, measuring the sensor’s responsiveness (^o^/RIU). **(B)** Detection accuracy (DA) as a function of virus concentration. **(C)** Quality factor (QF) (RIU^−1^), assessing the sharpness and reliability of the resonance dip. **(D)** Figure of Merit (FoM) (RIU^−1^), quantifying the sensor’s effectiveness. **(E)** Limit of Detection (LoD), indicating the minimum detectable viral concentration. **(F)** Signal-to-Noise Ratio (SNR), evaluating detection reliability.

The performance evaluation of the optimized Sys₄ and Sys₅ biosensors against SARS-CoV-2, as presented in Figures 8A–C and Table 3, provides valuable insights into their efficiency in detecting viral presence at different concentrations. By analyzing sensitivity to refractive index change (S, Equation 9), detection accuracy (DA, Equation 10), and quality factor (QF, Equation 11), we can assess their practical suitability for clinical applications. The sensitivity to refractive index change, shown in Figure 8A, highlights the superior optical response of Sys₅ compared to Sys₄. Sys₄ exhibits a sensitivity increase from 179.56°/RIU at 0.01 nM to 180.30°/RIU at 100 nM, whereas Sys₅ demonstrates a larger enhancement, ranging from 196.53°/RIU to 197.70°/RIU over the same concentration range. The consistently higher values in Sys₅ confirm that functionalizing the surface with ssDNA significantly improves sensitivity, as it facilitates stronger biomolecular interactions with the viral RNA, leading to more pronounced refractive index shifts. This increased sensitivity is essential for early-stage viral detection, as even minor concentration changes can be effectively captured.

**TABLE 3:** Numerical results of the optimized Sys₄ and Sys₅ biosensors for SARS-CoV-2 detection at different viral concentrations (0.01 nM–100 nM). The findings present the calculated Refractive Index (RI) of PBS + SARS-CoV-2 at nM concentrations, the resonance angle shift (Δθ), sensitivity to refractive index change (S, °/RIU), detection accuracy (DA, 10⁻^2^), and quality factor (QF, RIU⁻^1^).

Concentration (nM)	RI: PBS + SARS-CoV-2	∆θ	*S* ( °/RIU )	DA (10^−2^)	QF (*RIU* ^−1^)
Sys_4_					
0.01	1.3347306664113630	0.131	179.562	2.517	34.445
0.1	1.3347311385238432	0.131	179.446	2.517	34.423
1.0	1.3347358596486474	0.132	179.382	2.532	34.411
10	1.3347830708966875	0.140	179.294	2.693	34.393
50	1.3349928986657549	0.178	179.676	3,421	34.459
100	1.3355174680884236	0.274	180.300	5.244	34.558
Sys_5_					
0.01	1.3347306664113630	0.144	196.533	2.513	34.389
0.1	1.3347311385238432	0.144	196.406	2.513	34.366
1.0	1.3347358596486474	0.145	196.777	2.534	34.431
10	1.3347830708966875	0.154	196.662	2.694	34.405
50	1.3349928986657549	0.196	196.999	3.420	34.440
100	1.3355174680884236	0.300	197.698	5.236	34.503

The detection accuracy (DA), presented in Figure 8B, quantifies how precisely each biosensor differentiates viral-induced refractive index shifts. The results in Table 3 show that Sys₅ exhibits superior DA, increasing from 2.52 × 10⁻^2^ at 0.1 nM to 5.24 × 10⁻^2^ at 100 nM, while Sys₄ shows same trend. The fact that Sys₅ consistently maintains higher DA values suggests that the presence of ssDNA enhances selective binding to SARS-CoV-2 RNA, reducing the likelihood of false signals and improving diagnostic accuracy. The quality factor (QF), illustrated in Figure 8C, assesses the sharpness and stability of the SPR resonance peak, which is critical for reliable virus detection. Sys₄ maintains a stable QF of approximately 34.45 RIU⁻^1^ at 0.01 nM, increasing to 34.56 RIU⁻^1^ at 100 nM, whereas Sys₅ shows a slight variation from 34.39 RIU⁻^1^ to 34.50 RIU⁻^1^ over the same range. These results confirm that Sys₅ achieves improved biosensing performance while maintaining a well-defined resonance peak, ensuring stable and high-resolution virus detection.

We continue the performance evaluation of the optimized Sys₄ and Sys₅ biosensors, focusing on the Figure of Merit (FoM, Equation 12), Limit of Detection (LoD, Equation 13), and Signal-to-Noise Ratio (SNR, Equation 14), providing additional insight into their suitability for SARS-CoV-2 detection. Figures 8D–F and Table 4 summarize these critical parameters, which quantify the biosensor’s effectiveness, detection capability, and signal clarity. The Figure of Merit (FoM), displayed in Figure 8D, indicates the efficiency of the biosensor in detecting minute refractive index variations. Sys₄ consistently demonstrates significantly higher FoM values, beginning at 692.42 RIU⁻^1^ at 0.01 nM and gradually decreasing to 657.04 RIU⁻^1^ at 100 nM. In contrast, Sys₅ exhibits notably lower values, ranging from 232.25 RIU⁻^1^ at 0.01 nM to 218.76 RIU⁻^1^ at 100 nM. This considerable difference stems from Sys₄'s sharper resonance dip, which enhances its optical resolution. However, while Sys₄ maintains superior FoM values, the trade-off lies in its lower sensitivity to direct biomolecular interactions, as it lacks the ssDNA functionalization present in Sys₅.

**TABLE 4:** Additional performance metrics for optimized Sys₄ and Sys₅ biosensors at varying SARS-CoV-2 concentrations (0.01 nM–100 nM). The table presents the Figure of Merit (FoM, RIU⁻^1^), Limit of Detection (LoD, 10⁻⁵), and Signal-to-Noise Ratio (SNR).

Concentration (nM)	FoM (*RIU* ^−1^)	LoD (10^−5^)	SNR
Sys_4_			
0.01	692.424	2.785	0.025
0.1	691.953	2.786	0.025
1.0	691.473	2.787	0.025
10	688.812	2.788	0.027
50	680.003	2.782	0.034
100	657.042	2.773	0.052
Sys_5_			
0.01	232.246	2.544	0.025
0.1	232.087	2.545	0.025
1.0	232.434	2.541	0.025
10	231.389	2.542	0.027
50	227.772	2.538	0.034
100	218.759	2.529	0.052

The Limit of Detection (LoD), shown in Figure 8E, quantifies the minimum detectable viral concentration. Lower LoD values indicate a more sensitive sensor, capable of detecting smaller analyte concentrations. As observed in Table 4, Sys₄ maintains a LoD around 2.79 × 10⁻^5^ at 0.01 nM and decreases slightly to 2.77 × 10⁻^5^ at 100 nM, while Sys₅ exhibits slightly lower LoD values, starting at 2.54 × 10⁻^5^ and reaching 2.53 × 10⁻^5^ at 100 nM. The marginally lower LoD values of Sys₅ reinforce the importance of ssDNA functionalization, which contributes to enhanced biomolecular selectivity and improved interaction with viral RNA, allowing the system to detect lower concentrations of SARS-CoV-2. The Signal-to-Noise Ratio (SNR), presented in Figure 8F, assesses detection reliability by measuring signal clarity against background noise. Sys₄ consistently maintains higher SNR values, increasing from 0.0 at 0.01 nM to 0.05 at 100 nM, whereas Sys₅ follows the same trend. The higher SNR in Sys₄ and Sys₅ is expected due to its sharper resonance peaks, resulting in a clearer and more distinguishable optical signal. However, while Sys₄ benefits from stronger peak definition, it lacks the biorecognition capability provided by the ssDNA layer in Sys₅, which is a critical feature in direct viral detection.

### 3.9 Comparison with literature

The comparison presented in Table 5 highlights the advantages of the proposed MoSe₂-based SPR biosensors (Sys₄ and Sys₅) over previously reported designs for SARS-CoV-2 detection, particularly in terms of operating within clinically relevant concentration ranges and achieving high sensitivity. Most prior works, such as the ssDNA/Black Phosphorous/Si₃N₄/Ag/BK7 sensor, operate at high viral concentrations (150–525 mM), making them impractical for clinical diagnostics, where early-stage infection detection requires sensitivities in the nanomolar (nM) range. The CaF₂/Cu/BP/Graphene sensor achieves an impressive sensitivity of 410°/RIU within a 0–1000 nM concentration range, but it lacks the integration of a biorecognition layer (such as ssDNA), which limits its ability to directly interact with SARS-CoV-2 RNA. Similarly, the ssDNA/MoS₂/Si₃N₄/Ag/BK7 sensor demonstrates an improved sensitivity of 200°–261.3°/RIU, but it primarily operates at 1–150 mM concentrations, which may still be too high for detecting early-stage infections.

**TABLE 5:** Comparison of the proposed MoSe₂-based SPR biosensors (Sys₄ and Sys₅) with previously reported SPR sensor configurations for SARS-CoV-2 detection.

SPR sensor configuration	Testing concentration	Sensitivity ( °/RIU )	Ref.
ssDNA/Black Phorporous/Si_3_N_4_/Ag/BK7	150–525 mM	127–152	Kumar et al. (2022)
CaF_2_/Cu/BP/Graphene	0–1000 nM	410	Akib et al. (2024)
ssDNA/MoS_2_/Si_3_N_4_/Ag/BK7	1–150 mM	200–261.3	Tene et al. (2024b)
MoSe_2_/Si_3_N_4_/Ag/BK7	0.01–100 nM	179.6–180.3	This work
ssDNA/MoSe_2_/Si_3_N_4_/Ag/BK7	0.01–100 nM	196.5–197.7	This work

In contrast, our MoSe₂-based biosensors (Sys₄ and Sys₅) are specifically designed to detect SARS-CoV-2 at clinically relevant concentrations (0.01–100 nM), aligning with the viral load range found in patient samples. The sensitivity of Sys₄ (MoSe₂/Si₃N₄/Ag/BK7) ranges from 179.6°–180.3°/RIU, whereas Sys₅ (ssDNA/MoSe₂/Si₃N₄/Ag/BK7) achieves a superior sensitivity of 196.5°–197.7°/RIU, highlighting the crucial role of ssDNA functionalization in improving biomolecular interaction and enhancing specificity for SARS-CoV-2 detection. These findings reinforce the significance of combining 2D materials like MoSe₂ with a biorecognition layer (ssDNA) to improve biosensing performance. While other biosensors may exhibit higher sensitivity, they often operate in unrealistic high-concentration regimes, limiting their practical applicability.

Recent advancements in optical biosensing have introduced diverse approaches, including dielectric gratings, subwavelength gratings, and bimodal waveguide sensors, each offering high sensitivity and specificity. These technologies complement our MoSe₂-based SPR biosensors, demonstrating the versatility of photonic detection. Ref (Toma et al., 2024). reports the study on dielectric grating-based sensing, highlighting the enhanced spectral resolution, aligning with our goal of optimizing SPR biosensors for clinical applications. Similarly, Ref (Puumala et al., 2022). reports the study on silicon photonic biosensors with sub-wavelength gratings, improving optical confinement, comparable to the role of MoSe₂ and Si₃N₄ in our SPR biosensor design. Meanwhile, Ref (Torrijos-Morán et al., 2020). reports the study on bimodal waveguide grating sensors, achieving high bulk sensitivity (1350 nm/RIU), demonstrating another effective detection method.

While these alternative technologies provide unique advantages, our MoSe₂-based SPR biosensors offer a robust platform for SARS-CoV-2 detection at clinically relevant concentrations. The integration of MoSe₂ and Si₃N₄ achieves a strong balance between sensitivity, stability, and real-world applicability, positioning our approach as a viable contender among cutting-edge optical biosensors.

## 4 Conclusion

In this study, we developed and optimized a MoSe₂-based SPR biosensor for the detection of SARS-CoV-2, leveraging a numerical modeling approach based on the TMM. The biosensor’s structural parameters were systematically refined, focusing on key layers, including silver (Ag), silicon nitride (Si₃N₄), molybdenum diselenide (MoSe₂), and thiol-tethered single-stranded DNA (ssDNA), to enhance sensitivity, resonance stability, and molecular specificity. The optimization process demonstrated that a 45 nm Ag layer, 10 nm Si₃N₄ layer, and monolayer MoSe₂ configuration provided an optimal balance between attenuation, resonance sharpness (FWHM), and sensitivity enhancement. Furthermore, the inclusion of a 10 nm ssDNA functionalization layer significantly improved biomolecular interaction, leading to enhanced specificity for SARS-CoV-2 RNA detection at nM scale.

The optimized biosensor configurations, Sys₄ (MoSe₂-based) and Sys₅ (MoSe₂ + ssDNA functionalized), were evaluated under realistic viral concentrations (0.01 nM–100 nM). The results indicated that Sys₅ consistently outperformed Sys₄, exhibiting a resonance shift (Δθ) of 0.3° at 100 nM, a sensitivity of 197.70°/RIU, and a detection accuracy of 5.24 × 10⁻^2^, while maintaining a low limit of detection (LoD) of 2.53 × 10⁻^5^. Additionally, the quality factor (QF) and signal-to-noise ratio (SNR) remained within optimal ranges, confirming the biosensor’s robustness for realistic applications. While Sys₄ demonstrated superior optical resolution, its lack of a biorecognition layer limited its selectivity, reinforcing the necessity of ssDNA integration for direct RNA detection. These findings establish MoSe₂-based SPR biosensing as a promising candidate for rapid and precise SARS-CoV-2 detection, aligning with current advancements in plasmonic biosensor technology.

To further enhance the practical applicability of this work, experimental validation should be pursued to confirm the numerical predictions and assess fabrication feasibility. Additionally, integration with microfluidic platforms could enable real-time sensing in clinical settings. The incorporation of other 2D materials (e.g., WS₂, MoS₂, or graphene hybrids) may further improve optical response and detection efficiency. Finally, extending the biosensor framework to detect other respiratory viruses beyond SARS-CoV-2 could enhance the versatility of this sensing platform, making it suitable for broader pandemic preparedness efforts.

## Data Availability

The original contributions presented in the study are included in the article/Supplementary Material, further inquiries can be directed to the corresponding authors.
