# Supplementary material for: Mathematical modeling of a MoSe₂-based SPR biosensor for detecting SARS-CoV-2 at nM concentrations
Source: Front Bioeng Biotechnol. 2025 Feb 28;13:1547248. doi: 10.3389/fbioe.2025.1547248 (PMC11907102; doi:10.3389/fbioe.2025.1547248)
Supplement: Supplementary file 1 [file DataSheet1.docx]

Mathematical Modeling of a MoSe₂-based SPR Biosensor for Detecting the Novel Coronavirus

Talia Tene^1^, Nataly Bonilla García^2^, Jessica Alexandra Marcatoma Tixi^3^, Martha Ximena Dávalos Villegas^4^, Cristian Vacacela Gomez^5^, Stefano Bellucci^5,^*

^1^Department of Chemistry, Universidad Técnica Particular de Loja, Loja 110160, Ecuador

^2^Escuela Superior Politécnica de Chimborazo (ESPOCH), Riobamba 060155, Ecuador

^3^Carrera de Estadística, Escuela Superior Politécnica de Chimborazo (ESPOCH), Riobamba 060155, Ecuador

^4^Carrera de Matemática, Escuela Superior Politécnica de Chimborazo (ESPOCH), Riobamba 060155, Ecuador

^5^INFN-Laboratori Nazionali di Frascati, Via E. Fermi 54, 00044 Frascati, Italy

***** Correspondence:

[tbtene@utpl.edu.ec](mailto:tbtene@utpl.edu.ec) (Talia Tene)

[vacacela@lnf.infn.it](mailto:vacacela@lnf.infn.it) (Cristian Vacacela Gomez)

**Supplementary Figures**

**
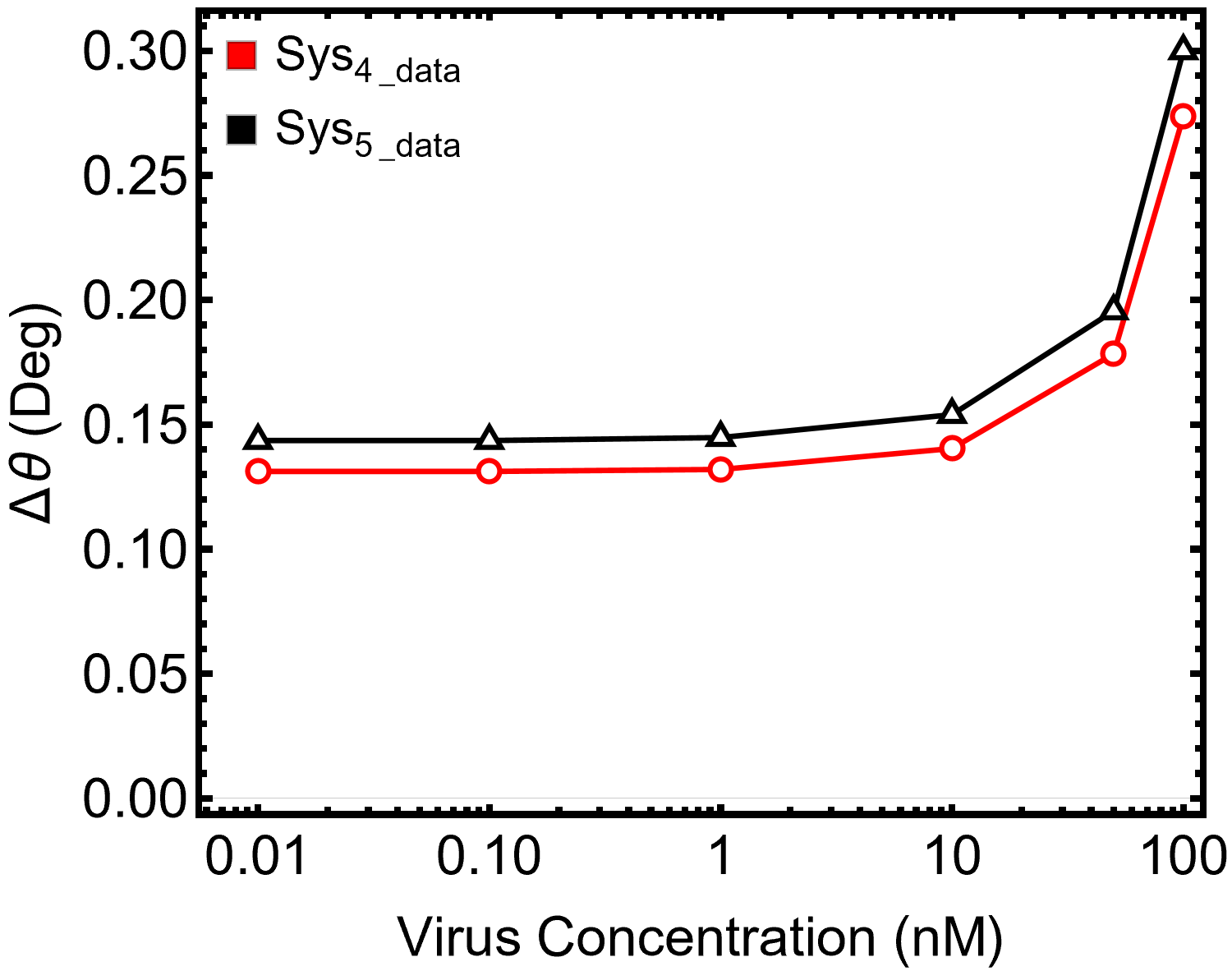
**

**Figure S1.** Resonance angle shift (Δθ) variation for Sys₄ and Sys₅ after SARS-CoV-2 sensing at different viral concentrations (0.01 nM to 100 nM), relative to their respective PBS-only baselines ([PBS@0.0__Sys4_](mailto:PBS@0.0_Sys4) and PBS@0.0__Sys5_). The results indicate that Sys₅ exhibits a larger angle shift across all concentrations, suggesting higher sensitivity and better refractive index response compared to Sys₄.

**Supplementary Tables**

**Table S1.** Initial material parameters used for the MoSe₂-based SPR biosensor design. The table includes the refractive index (RI) at 633 nm and the corresponding layer thicknesses (nm) for each material in the sensor structure. The refractive indices of BK-7 prism, Silver (Ag), Silicon Nitride (Si₃N₄), MoSe₂, Thiol-Tethered ssDNA (T), Water, PBS medium, and SARS-CoV-2 are provided, along with their respective theoretical/experimental references.

| **Material** | **Refractive Index at 633 nm** | **Thickness (nm)** | **Ref. #** |
| --- | --- | --- | --- |
| BK-7 (P) | 1.5151 | --- | Typical value used |
| Silver (Ag) | 0.056253 + 4.2760 i | 55.0 | [23] |
| Si_3_N_4_ (SN) | 2.0394 | 5.00 | [29] |
| Molibdene Diselenide (MoSe_2_) | 4.62 + 1.0063 i | 0.70 | [31] |
| ssDNA (Thiol-Tethered, T) | 1.462 | 3.20 | [29] |
| Water medium (H_2_O) | 1.330 | --- | Typical value used |
| PBS medium | 1.334 | --- | Typical value used |
| SARS-CoV-2 | [1.340@150](mailto:1.340@150) mM | --- | [29] |

**Table S2.** Numerical values of key optical parameters for each SPR biosensor configuration (Sys₀ to Sys₅). Sys₀ represents the most basic system in PBS, while Sys₁ is the simplest system in PBS + Virus. From Sys₂ to Sys₅, additional layers (Si₃N₄, MoSe₂, and ssDNA) are introduced under PBS + Virus conditions. The table provides the SPR peak position (°), attenuation percentage (%), Full Width at Half Maximum (FWHM, °), and sensitivity enhancement (%) compared to Sys₀. Sys₅ exhibits the highest enhancement, confirming its superior sensing performance.

| **Sys No.** | **Code** | **SPR Peak position** | **Attenuation (%)** | **FWHM** | **Enhancement (%)** |
| --- | --- | --- | --- | --- | --- |
| 0 | Sys_0_ | 67.941 | 0.021 | 0.898 | 0.00 |
| 1 | Sys_1_ | 68.651 | 0.019 | 0.936 | 1.044 |
| 2 | Sys_2_ | 71.288 | 0.004 | 1.291 | 4.926 |
| 3 | Sys_3_ | 71.791 | 0.001 | 1.356 | 5.665 |
| 4 | Sys_4_ | 72.755 | 17.334 | 2.694 | 7.086 |
| 5 | Sys_5_ | 73.344 | 17.640 | 2.828 | 7.951 |

**Table S3.** Numerical values of key optical parameters for each SPR biosensor configuration by optimizing the silver thickness

| **Thickness (nm)** | **SPR Peak position** | **Attenuation (%)** | **FWHM** | **Enhancement (%)** |
| --- | --- | --- | --- | --- |
| **Sys_4_** | | | | |
| 40 | 72.589 | 5.272 | 4.317 | 1.007 |
| 45 | 72.660 | 0.003 | 3.534 | 1.106 |
| 50 | 72.715 | 4.677 | 3.015 | 1.182 |
| 55 | 72.755 | 17.334 | 2.695 | 1.238 |
| 60 | 72.784 | 33.781 | 2.525 | 1.278 |
| 65 | 72.804 | 50.054 | 2.483 | 1.306 |
| **Sys_5_** | | | | |
| 40 | 73.136 | 5.149 | 4.444 | 0.953 |
| 45 | 73.226 | 0.001 | 3.671 | 1.077 |
| 50 | 73.294 | 4.845 | 3.151 | 1.171 |
| 55 | 73.343 | 17.640 | 2.828 | 1.240 |
| 60 | 73.378 | 34.144 | 2.656 | 1.288 |
| 65 | 73.402 | 50.402 | 2.613 | 1.321 |

**Table S4.** Numerical values of key optical parameters for each SPR biosensor configuration by optimizing the silicon nitride thickness

| **Thickness (nm)** | **SPR Peak position** | **Attenuation (%)** | **FWHM** | **Enhancement (%)** |
| --- | --- | --- | --- | --- |
| **Sys_4_** | | | | |
| 5 | 72.660 | 0.003 | 3.616 | 1.202 |
| 10 | 77.125 | 0.733 | 5.111 | 7.422 |
| 15 | 84.710 | 20.259 | 7.351 | 17.986 |
| 20 | 83.793 | 90.626 | 14.625 | 16.708 |
| 25 | 80.783 | 96.106 | 249.285 | 12.517 |
| 30 | 77.398 | 97.060 | 25.290 | 7.801 |
| **Sys_5_** | | | | |
| 5 | 73.226 | 0.001 | 3.772 | 1.199 |
| 10 | 77.982 | 1.033 | 5.334 | 7.772 |
| 15 | 85.658 | 36.287 | 7.789 | 18.381 |
| 20 | 83.379 | 92.362 | 16.303 | 15.231 |
| 25 | 80.316 | 96.379 | 121.811 | 10.997 |
| 30 | 76.858 | 97.131 | 61.460 | 6.218 |

**Table S5.** Numerical values of key optical parameters for each SPR biosensor configuration by optimizing the number of Molybdenum Diselenide layers

| **Layers** | **SPR Peak position** | **Attenuation (%)** | **FWHM** | **Enhancement (%)** |
| --- | --- | --- | --- | --- |
| **Sys_4_** | | | | |
| L1 | 77.126 | 0.733 | 5.190 | 1.462 |
| L2 | 79.993 | 14.857 | 7.724 | 5.243 |
| L3 | 82.693 | 40.186 | 9.853 | 8.785 |
| L4 | 83.107 | 65.183 | 11.581 | 9.331 |
| L5 | 82.419 | 78.143 | 13.092 | 8.426 |
| L6 | 81.637 | 84.393 | 14.294 | 7.396 |
| **Sys_5_** | | | | |
| L1 | 77.982 | 1.033 | 5.398 | 1.485 |
| L2 | 81.042 | 17.571 | 7.959 | 5.468 |
| L3 | 83.341 | 46.959 | 10.052 | 8.459 |
| L4 | 83.164 | 70.209 | 11.837 | 8.229 |
| L5 | 82.341 | 80.820 | 13.342 | 7.159 |
| L6 | 81.539 | 85.942 | 14.505 | 6.114 |

**Table S6.** Numerical values of key optical parameters for each SPR biosensor configuration by optimizing the ssDNA thickness

| **Thickness (nm)** | **SPR Peak position** | **Attenuation %** | **FWHM** | **Enhancement (%)** |
| --- | --- | --- | --- | --- |
| **Sys_5_** | | | | |
| 3.2 | 77.983 | 1.034 | 5.334 | 1.487 |
| 5.0 | 78.483 | 1.265 | 5.462 | 2.138 |
| 10.0 | 79.955 | 2.293 | 5.824 | 4.054 |
| 20.0 | 83.359 | 9.139 | 6.612 | 8.484 |
| 30.0 | 86.056 | 44.711 | 7.665 | 11.994 |
| 50.0 | 84.549 | 89.732 | 13.138 | 10.032 |

**Table S7.** Numerical values of key optical parameters for each SPR biosensor configuration by varying the viral concentration in nM scale

| **Concentration (nM)** | **RI: PBS + SARS-CoV-2** | **SPR Peak position** | **Attenuation %** | **FWHM** | **Enhancement (%)** |
| --- | --- | --- | --- | --- | --- |
| **Sys_4_** | | | | | |
| 0.01 | 1.3347306664113630 | 0.0 | 0.0 | 0.0 | 0.0 |
| 0.1 | 1.3347311385238432 | 78.69 | 0.497 | 5.212 | 0.172 |
| 1.0 | 1.3347358596486474 | 78.69 | 0.497 | 5.213 | 0.173 |
| 10 | 1.3347830708966875 | 78.70 | 0.499 | 5.213 | 0.184 |
| 50 | 1.3349928986657549 | 78.75 | 0.507 | 5.214 | 0.234 |
| 100 | 1.3355174680884236 | 78.86 | 0.526 | 5.217 | 0.359 |
| **Sys_5_** | | | | | |
| 0.01 | 1.3347306664113630 | 0.0 | 0.0 | 0.0 | 0.0 |
| 0.1 | 1.3347311385238432 | 83.16 | 1.481 | 5.715 | 0.182 |
| 1.0 | 1.3347358596486474 | 83.16 | 1.481 | 5.715 | 0.184 |
| 10 | 1.3347830708966875 | 83.17 | 1.487 | 5.716 | 0.196 |
| 50 | 1.3349928986657549 | 83.23 | 1.512 | 5.719 | 0.249 |
| 100 | 1.3355174680884236 | 83.40 | 6.30 | 5.730 | 0.55 |
